# Cryo-EM reconstruction of the human 40S ribosomal subunit at 2.15 Å resolution

**DOI:** 10.1093/nar/gkad194

**Published:** 2023-03-23

**Authors:** Simone Pellegrino, Kyle C Dent, Tobias Spikes, Alan J Warren

**Affiliations:** Department of Haematology, University of Cambridge, Hills Road, Cambridge CB2 0XY, UK; Cambridge Institute for Medical Research, Keith Peters Building, Hills Road, Cambridge CB2 0XY, UK; Wellcome Trust–Medical Research Council Stem Cell Institute, University of Cambridge, Cambridge, UK; Department of Haematology, University of Cambridge, Hills Road, Cambridge CB2 0XY, UK; Cambridge Institute for Medical Research, Keith Peters Building, Hills Road, Cambridge CB2 0XY, UK; Wellcome Trust–Medical Research Council Stem Cell Institute, University of Cambridge, Cambridge, UK; Department of Haematology, University of Cambridge, Hills Road, Cambridge CB2 0XY, UK; Cambridge Institute for Medical Research, Keith Peters Building, Hills Road, Cambridge CB2 0XY, UK; Wellcome Trust–Medical Research Council Stem Cell Institute, University of Cambridge, Cambridge, UK; Department of Haematology, University of Cambridge, Hills Road, Cambridge CB2 0XY, UK; Cambridge Institute for Medical Research, Keith Peters Building, Hills Road, Cambridge CB2 0XY, UK; Wellcome Trust–Medical Research Council Stem Cell Institute, University of Cambridge, Cambridge, UK

## Abstract

The chemical modification of ribosomal RNA and proteins is critical for ribosome assembly, for protein synthesis and may drive ribosome specialisation in development and disease. However, the inability to accurately visualise these modifications has limited mechanistic understanding of the role of these modifications in ribosome function. Here we report the 2.15 Å resolution cryo-EM reconstruction of the human 40S ribosomal subunit. We directly visualise post-transcriptional modifications within the 18S rRNA and four post-translational modifications of ribosomal proteins. Additionally, we interpret the solvation shells in the core regions of the 40S ribosomal subunit and reveal how potassium and magnesium ions establish both universally conserved and eukaryote-specific coordination to promote the stabilisation and folding of key ribosomal elements. This work provides unprecedented structural details for the human 40S ribosomal subunit that will serve as an important reference for unravelling the functional role of ribosomal RNA modifications.

## INTRODUCTION

The ribosome is a ribonucleoprotein machine that translates the genetic code in every living organism. It consists of two subunits, comprising 4 ribosomal RNAs (rRNAs) and 80 ribosomal proteins (RPs). The large (60S) subunit is responsible for peptide bond formation, while the small (40S) subunit reads and decodes the messenger RNA (mRNA). Assembly of ribosomal subunits is one of the most energy consuming processes within cells, requiring >300 biogenesis factors and 80 small nucleolar RNAs (snoRNAs) in humans (1,2). Similarly, protein synthesis is a tightly regulated process that involves the sequential action of translation factors to ensure speed and accuracy of the process (3).

The rRNAs and RPs are decorated with several types of modifications. Post-transcriptional modifications are mainly incorporated early in the process of ribosome biogenesis, likely soon after the primary 47S transcript is generated (2–4). There are 14 distinct rRNA modifications within the human 80S ribosome, which decorate approximately 5% of rRNA residues. These modifications are thought to stabilise rRNA structure and participate in the binding of functional ligands during ribosome biogenesis and translation (2,5,6). Eukaryotic rRNA modifications mainly comprise 2’-*O*-methylation of rRNA ribose and the isomerisation of the nucleoside uridine to pseudouridine (psi, ψ); these chemical modifications are introduced by box C/D and box H/ACA snoRNPs, respectively, with nucleotide specificity dictated by the sequence complementarity of snoRNAs, which pair with the target site (7,8). All the other rRNA modifications involve only nucleotide bases and are introduced by stand-alone enzymes that function throughout the ribosome biogenesis pathway (8). Distinct patterns of rRNA modifications may promote ribosome specialisation, promoting the selective translation of subsets of mRNAs (9–11). Furthermore, rRNA modifications may also be dysregulated in cancer (12–19). Methods such as 2D thin layer chromatography, primer extension-based assays, or chemical derivatisation were initially used to identify 2′-*O*-methylation and pseudouridylation sites (20–23). More recently, ‘stable isotope–labelled ribonucleic acid as an internal standard’ (SILNAS)-based quantification of post-transcriptional modifications (6) and cryo-electron microscopy (cryo-EM) (5) approaches have sought to identify the complete repertoire of modifications on the human 80S ribosome. However, given the discrepancies in the published data between these methods, higher resolution structures of human ribosomes are required to directly visualise rRNA modifications with greater accuracy to better resolve their function.

Here, we report a cryo-EM reconstruction of the human 40S ribosomal subunit at an overall resolution of 2.15 Å, extending to 1.8 Å in the core, revealing unprecedented structural details. We visualise post-transcriptional modifications incorporated into the 18S rRNA of the 40S ribosomal subunit and identify experimental density for post-translational modifications of ribosomal proteins. Furthermore, we model the solvation shells of the 40S ribosomal subunit, revealing the position of monovalent and divalent cations.

## MATERIALS AND METHODS

### Purification of human 40S ribosomal subunits

To purify human 40S ribosomal subunits, we adapted a previously published protocol used for yeast ribosomes (24). Human Embryonic Kidney Expi293 cells were grown to a density of approximately 2 × 10^6^ in Expi293 medium (Thermo Scientific). Following stress induction upon addition of 100 mM (final concentration) NaCl to 250 ml of culture for 20 min, cells were harvested by centrifugation at 800 × g for 10 min. After two consecutive washes with phosphate buffer saline (PBS), cells were harvested by centrifugation, flash frozen in liquid N_2_ and stored at −80°C. Cells were thawed on ice for 30 min and later resuspended in lysis buffer (30 mM HEPES–KOH pH 7.5, 50 mM KCl, 10 mM MgCl_2_, 220 mM sucrose, 2 mM DTT, 0.5 mM EDTA, supplemented with 0.5 mM NaF, 0.1 mM Na_3_VO_4_, 0.5× protein inhibitors cocktail tablet (Roche), 2000 units of RNasin Plus ribonuclease inhibitor (Promega), 0.5 mg/mL heparin and 0.5% Igepal-630 (Sigma)). The slurry was incubated for 30 min on ice with periodic mixing, then centrifuged at 10 000 × g for 5 min at 4°C. The recovered soluble fraction was centrifuged at 30 000 × g for 20 min at 4°C. The resulting supernatant was placed in a separate tube, incubated on ice for 15 min and following the addition of PEG 20 000 to a final concentration of 1.35%, centrifuged at 20 000 × g for 12 min at 4°C. The supernatant was placed in a clean tube and the KCl raised to a final concentration of 130 mM. The solution was incubated for 5 min and cleared by centrifugation at 17 500 × g for 5 min. The concentration of PEG 20000 was raised to 4.25% and the solution incubated for 10 min in ice. After centrifugation at 17 500 × g for 10 min at 4°C the white ribosome-containing pellet was resuspended in 750 μl of buffer R (30 mM HEPES–KOH pH 7.5, 125 mM KCl, 7.5 mM MgCl_2_, 2 mM DTT containing 0.5 mg/ml heparin and 0.1× protease inhibitor cocktail) on ice. 80 OD_260_ were loaded per 10–30% sucrose gradient, prepared in buffer containing 25 mM HEPES–KOH pH 7.5, 125 mM KCl, 8.3 mM MgCl_2_, 0.5 mM EDTA, 2 mM DTT. Gradients were centrifuged at 42 800 × g for 15 h at 4°C in an SW28 rotor (Beckman Coulter). Fractions of 1 ml were collected using an AKTA system (Cytiva). Those fractions corresponding to the 80S peak were pooled and precipitated by adding PEG 20000 at a final concentration of 5.15%. The solution was incubated for 10 min at 4°C and centrifuged at 17 500 × g for 10 min at 4°C. The resulting pellet was resuspended in buffer S (25 mM HEPES–KOH pH 7.5, 100 mM KCl, 7.5 mM MgCl_2_ and 2 mM DTT). 70 OD_260_ were layered onto a 10–40% sucrose gradient prepared in buffer containing 25 mM HEPES pH 7.5, 550 mM KCl, 5 mM MgCl_2_ and 2 mM DTT and centrifuged at 70 000 × g for 20.5 h at 4°C in an SW28 rotor (Beckman Coulter). Fractions corresponding to the 40S peak were collected, pooled and 40S subunits precipitated by adding 7.5% of PEG 20000 (final concentration) for 10 min on ice and centrifuging at 17 500 × g for 10 min at 4°C. The final pellet was resuspended in buffer containing 20 mM HEPES–KOH pH7.5, 100 mM KCl, 5 mM MgOAc2 and 1 mM DTT. Aliquots (final concentration of 35 OD_260_/μl) were flash frozen in liquid N2 and stored at −80°C.

### Cryo-EM grids preparation, data collection and image processing

R2/2 holey carbon supported copper grids (Quantifoil) coated with a 2nm thin carbon layer were functionalised as described (25). In brief, prior sample deposition, grids were soaked for 30 s in 50 mM 1-pyrenemethylamine (Sigma-Aldrich #401633) and then washed 5 s in 1 ml of isopropanol and another 5 s in 1 ml of ethanol. After air drying the grids at RT for approximately 30 min, 4 μl of 40S sample (at a concentration of 2.5 OD_260_/μl) was directly applied onto each grid. The grid was incubated for 30 s at 4 °C and 95% humidity, then blotted for 1 s (blot force −7) prior to plunging into liquid ethane using a Vitrobot Mark IV (FEI Company). Grids were screened for ice quality and cryo-EM data was acquired on a Titan Krios transmission electron microscope (FEI Company) at 300 kV equipped with a K3 direct electron detector (Gatan). The dataset was recorded in counting super-resolution mode at a nominal magnification of 105 000× (corresponding to a pixel size of 0.83 Å/px on the object scale (0.415 Å in super-resolution) with a defocus range of −1.0 to −2.8 μm and a dose of ∼49 e^−^/Å^2^. Acquisition of 3091 movies was performed semi-automatically using EPU software (FEI Company) and pre-processed on-the-fly using Warp (26). The resulting set of picked particles was imported into cryoSPARC (27) to perform initial processing and remove non-ribosomal and/or damaged particles from the dataset. After *ab initio* reconstruction, heterogeneous and uniform refinement, a total of 474276 particles were imported into Relion 3.0 (28) for further processing. Original movies were corrected for the effects of beam-induced motion using MotionCor2 (29) as implemented in the Relion GUI, and particles from cryoSPARC re-extracted. A first round of Refine3D was performed using the map resulting from homogeneous refinement in cryoSPARC as a reference. The estimated resolution after postprocessing was 2.41 Å. CTF and beam tilt refinement followed by Bayesian polishing was repeated twice, and Refine3D performed after each step. Final postprocessing of the entire human 40S yielded a reconstruction at 2.15 Å resolution, with a B-factor of −35 Å^2^. The alignment of the particles is driven by the body domain of the 40S subunit, as the head is mobile. To achieve high-resolution reconstructions for both domains, two overlapping soft masks including the individual domains were created within Relion and used as inputs for multibody refinement (30). The two resulting reconstructions yielded a resolution estimation of 2.24 Å (*B*-factor: −39 Å^2^) for the head domain and 2.09 Å (*B*-factor: −33 Å^2^) for the body domain. All resolution estimations are based on the gold-standard Fourier Shell Correlation (FSC) criterion of 0.143 (31,32).

### Model building and validation

The resulting maps were used for model building in Coot (33) using the mature 40S ribosomal subunit (PDB: 6G5H) as a reference. Protein and rRNA chains were inspected in Coot and manually fitted into the density. To interpret regions at lower local resolution, we used LocalDeblur (34) from the Scipion suite (35) to generate locally sharpened maps of the head and the body domains. To add water molecules, we employed a semi-automatic approach using phenix.douse (Phenix suite, (36)) and visual inspection in Coot. Building of additional water molecules and ions, such as Mg^2+^ and K^+^, was performed manually in Coot, based on the coordination geometries. Models were refined using real space refinement as implemented in PHENIX (36) and validated using MolProbity (37).

### Mass spectrometry

A sample containing 30 OD_260_ of the human 40S subunit was loaded onto a 4–12% acrylamide gel and electrophoresed for 5 min to remove excess sucrose. Gel bands were excised, destained, reduced (using DTT), alkylated (iodoacetamide) and subjected to enzymatic digestion with trypsin (Promega) overnight at 37°C. After digestion, the supernatant was pipetted into a sample vial and loaded onto an autosampler for automated LC-MS/MS analysis. The liquid chromatography (LC)–MS/MS experiment was performed using a Dionex Ultimate 3000 RSLC nanoUPLC (Thermo Fisher Scientific) system and a Q Exactive Orbitrap mass spectrometer (Thermo Fisher Scientific). Separation of peptides was performed by reverse-phase chromatography at a flow rate of 300 nl/min and a Thermo Scientific reverse-phase nano Easy-spray column (Thermo Scientific PepMap C18, 2 μm particle size, 100 Å pore size, 75 μm i.d. × 50 cm length). Peptides were loaded onto a pre-column (Thermo Scientific PepMap 100 C18, 5 μm particle size, 100 Å pore size, 300 μm i.d. × 5 mm length) from the Ultimate 3000 autosampler with 0.1% formic acid for 3 min at a flow rate of 15 μl/min. After this period, the column valve was switched to allow elution of peptides from the pre-column onto the analytical column. Solvent A was water + 0.1% formic acid and solvent B was 80% acetonitrile, 20% water + 0.1% formic acid. The linear gradient employed was 2–40% B over 90 min (the total run time including column washing and re-equilibration was 120 min). The LC eluant was sprayed into the mass spectrometer by means of an Easy-spray source (Thermo Fisher Scientific Inc.). All *m/z* values of eluting ions were measured in an Orbitrap mass analyser, set at a resolution of 35 000 and scanned between *m/z* 380–1500. Data dependent scans (Top 20) were employed to automatically isolate and generate fragment ions by higher energy collisional dissociation (HCD). Normalised collision energy (NCE): 25%) in the HCD collision cell and measurement of the resulting fragment ions was performed in the Orbitrap analyser, set at a resolution of 17 500. Singly charged ions and ions with unassigned charge states were excluded from selection for MS/MS and a dynamic exclusion of 60 s was employed.

The files were submitted to the Mascot search algorithm (Matrix Science, London UK, version 2.6.0) and searched against a common contaminants database (125 sequences; 41 129 residues) and the UniProt human database (CCP_UniProt_homo sapiens_proteome_20180409 database, 93 609 sequences; 37 041 084 residues). Variable modifications of oxidation (M), deamidation (NQ) phosphorylation (S,T and Y) acetylation (K and protein N-terminus), methylation (HKNR), sulfation (STY) and ubiquitination (K) were applied together with a fixed modification of carbamidomethyl (C). The peptide and fragment mass tolerances were set to 20 ppm and 0.1 Da, respectively. A significance threshold value of *P* < 0.05 and a peptide cut-off score of 20 were also applied. Scaffold (version Scaffold_4.10.0, Proteome Software Inc., Portland, OR) was used to validate MS/MS based peptide and protein identifications. Peptide identifications were accepted if they could be established at >95.0% probability by the Peptide Prophet algorithm (38) with Scaffold delta-mass correction. Protein identifications were accepted if they could be established at greater than 99.0% probability and contained at least two identified peptides. Protein probabilities were assigned by the Protein Prophet algorithm (39). Proteins that contained similar peptides and could not be differentiated based on MS/MS analysis alone were grouped to satisfy the principles of parsimony. Proteins sharing significant peptide evidence were grouped into clusters.

## RESULTS

### Direct visualisation of human 40S rRNA modifications

**Figure 1.**
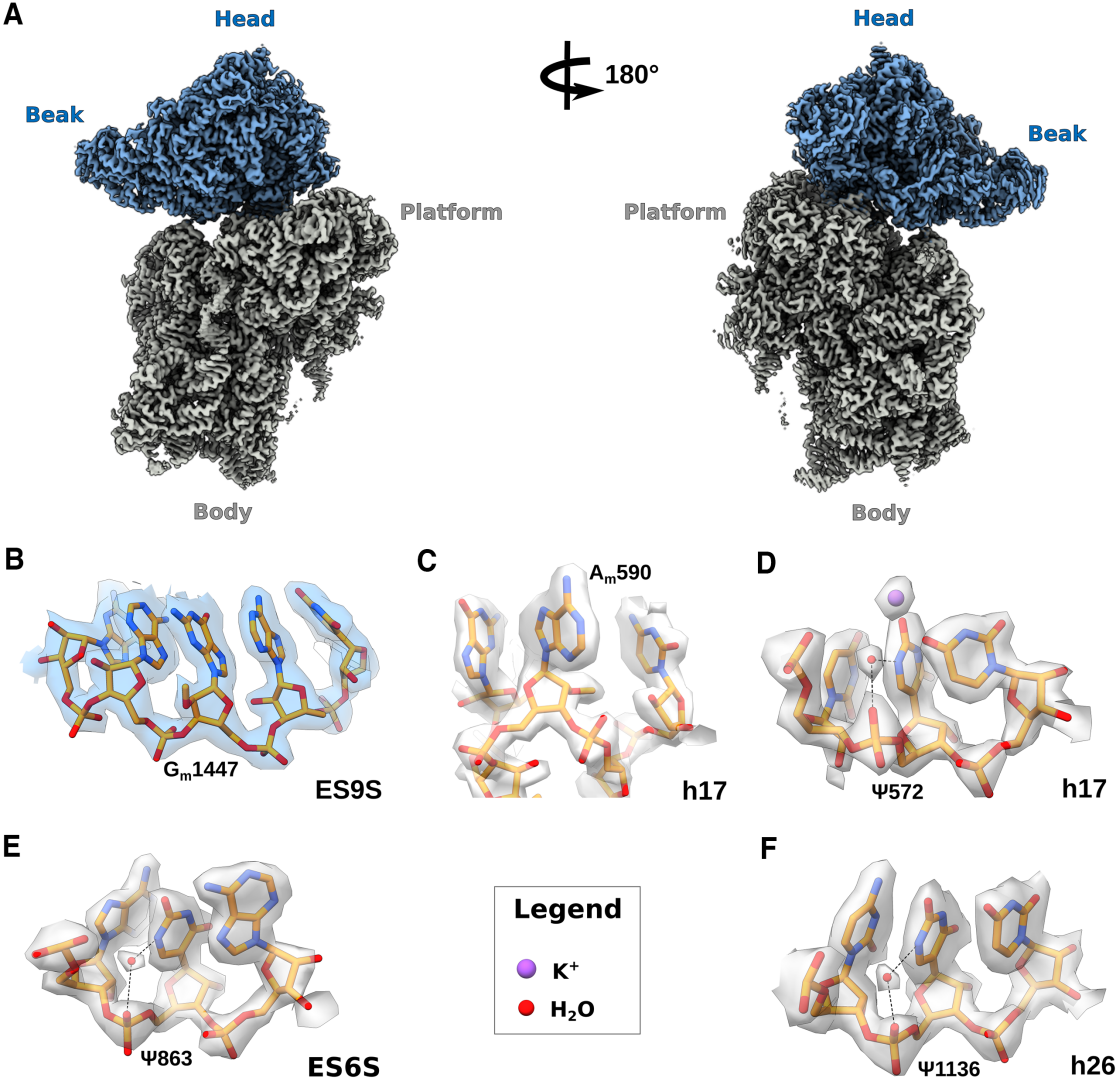
rRNA modifications of the human 40S subunit at 2.15 Å resolution. (**A**) Cryo-EM reconstruction of the human 40S ribosomal subunit viewed from the intersubunit interface (left) and rotated 180° (right). The map corresponding to the head domain is coloured blue, body in grey. Water molecules and K^+^ ions are shown as spheres (legend at the bottom of the panel). (**B**) 2’-*O*-Methylated G1447 in expansion segment 9S (ES9S) of h39. (**C**) Close-up of the tip of h17, showing 2’-*O*-methylated A590. (**D**) Uridine isomerisation at residue U572 in h17. (**E**) Uridine isomerisation at residue U863, within ES6S. (**F**) Uridine isomerisation at residue U1136, with a water molecule coordinating the imino group at position 1.

The small ribosomal subunit consists of two domains, the body and the head (Figure 1A, Supplementary Figure S1). The head is highly dynamic and rotates (‘swivels’) with respect to the main axis of the body when not interacting with functional ligands, such as transfer RNA (tRNA), mRNA, assembly or translation factors. The intrinsic mobility of these two domains and the presence of elements that are flexible when not interacting with the large ribosomal subunit, such as h44, have hampered efforts to achieve high-resolution reconstructions of the 40S ribosomal subunit. By employing a novel method for sample preparation (see Materials and Methods) and by implementing the multibody refinement algorithm in Relion 3.0 (30) (Supplementary Figures S1 and S2), we obtained individual reconstructions of both the head and body at nominal resolutions of 2.24 and 2.09 Å, respectively, extending to 1.8 Å resolution in the most stable regions (Figure 1A, Supplementary Figure S2, Supplementary Table S1). Regions of the 40S subunit that are intrinsically flexible have been modelled using a locally filtered map (see Materials and Methods).

**Figure 2.**
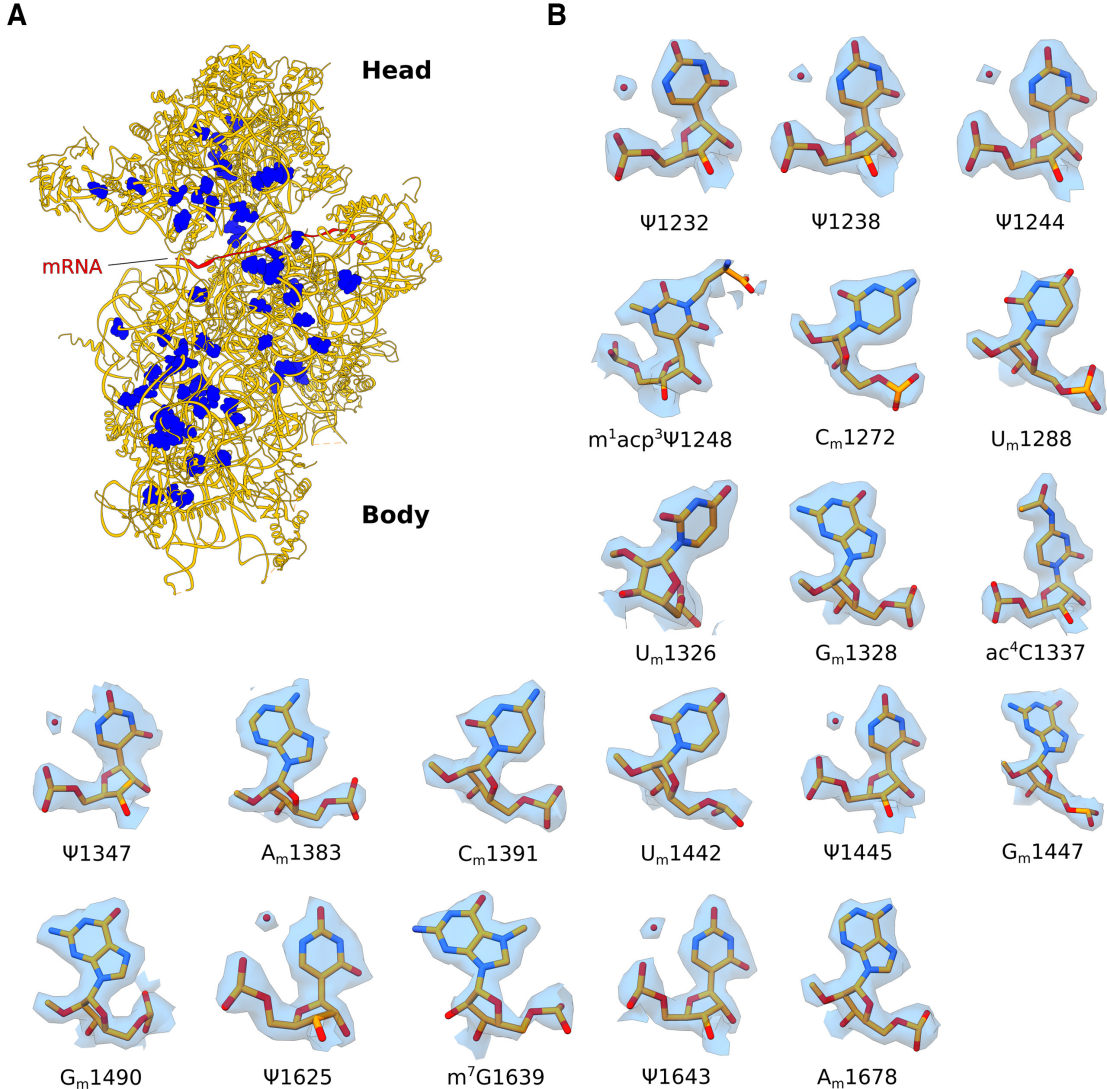
rRNA modifications of the head domain. (**A**) Ribbon representation of the human 40S subunit (orange) with rRNA modifications shown as blue spheres. For clarity, mRNA was modelled *in silico* by superposing the human 48S initiation complex (PDB: 6ZMW). (**B**) rRNA modifications visualised in this study fitted into the experimental cryo-EM map, for the head domain only. Water molecules (red spheres) coordinated with pseudouridine bases are indicated.

**Figure 3.**
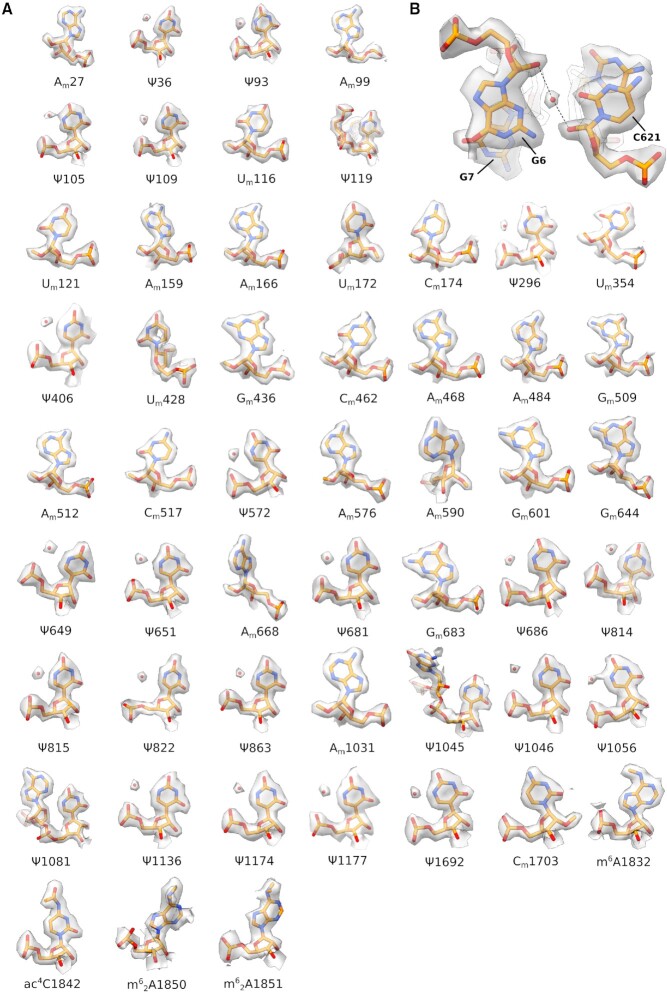
rRNA modifications in the body domain. (**A**) rRNA modifications within the body. Water molecules (red spheres) coordinated with pseudouridine bases are shown. (**B**) Detail of the universally conserved rRNA residue C621 (C525 bacterial numbering) on h18. A water molecule (red sphere) coordinated with the 2’-*O* of the sugar backbone of C621 is conserved between higher eukaryotes and prokaryotes (PDB: 7K00).

Our cryo-EM reconstructions allowed us to visualise unambiguously 73 out of the 91 rRNA modifications of the human 40S ribosomal subunit identified by SILNAS-based quantification (6) (Figures 2A, B and 3A, Supplementary Table S2). We visualised chemical modifications that were either previously unassigned (5) or were yet unmodelled in the human 40S ribosomal subunit, including 2’-*O*-methylation (rRNA residues G1447 and A590) (Figure 1B, C), and uridine isomerisation (residues U572 and U863) (Figure 1D, E, 2B, 3A). Our findings are in good overall agreement with data from 2OMet-seq (40), RiboMeth-seq (41), Pseudo-seq (42) and SILNAS-based analysis of rRNA modifications (6). Of the four new modifications identified by Taoka et al. (2’-*O*-methylation of residue C621 and pseudouridines ψ897, ψ1045, ψ1136 and ψ1232), we visualised three of the four proposed pseudouridines (ψ1045, ψ1136 and ψ1232), while ψ897 localises to a flexible region where the cryo-EM density could not be interpreted (Figures 1F, 2B and 3A, Supplementary Table S2). While the imino group at position 1 of ψ1045 directly contacts the 2’-OH group of the neighbouring G1044, the isomeric nucleobases of ψ1136 and ψ1242 establish water-mediated hydrogen bonds with the phosphate backbone. Interestingly, ψ1136 was present in low sub-stoichiometric amounts in human TK6 cells using SILNAS (6) but was not identified in previous cryo-EM reconstructions of either the human 80S ribosome (5) or late pre-40S subunits (43). However, our map revealed clear density for a water molecule mediating an interaction between the N1 imino group of uridine and the phosphate backbone (Figures 1F and 3A). By contrast, we did not identify 2’-*O*-methylation of residue C621, a modification that would likely disrupt water-mediated hydrogen bonding interactions between the two nucleobases of residues C621 and G6 (Figure 3B). Both the map and the atomic model of a human late pre-40S particle (PDB ID: 6ZXG) further support the absence of 2’-*O*-methylation of residue C621.

### Conserved and eukaryote-specific K^+^ ion binding sites

**Figure 4.**
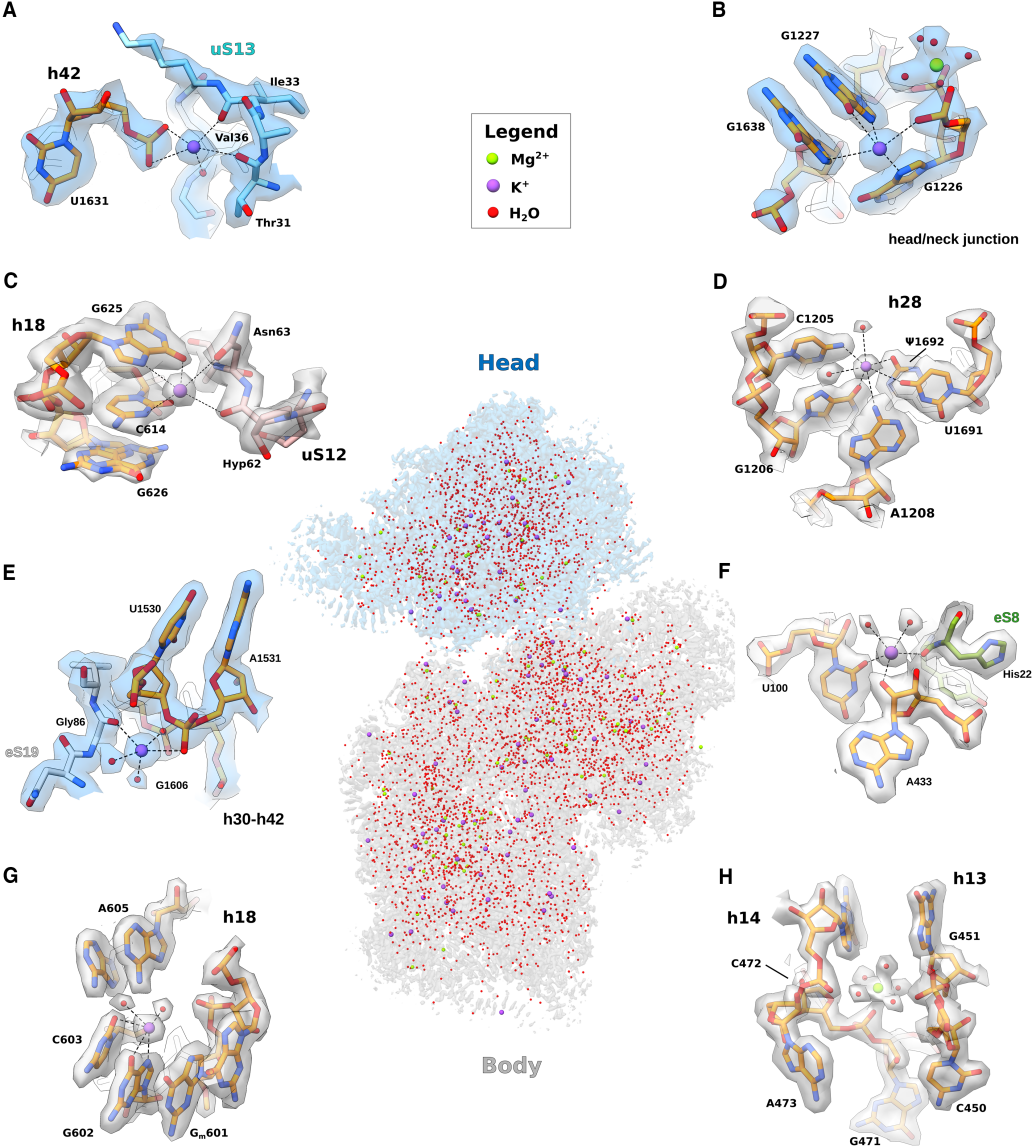
Solvation shell of the human 40S subunit. Middle of panel: map of the head (blue) and body (grey) domains; water molecules, K^+^ and Mg^2+^ ions are shown as spheres (legend at the top of the panel). (**A**) K^+^ ion bridges the universally conserved ribosomal protein uS13 and h42 within the head domain of the 18S rRNA. (**B**) K^+^ ion maintains rRNA planarity within the head/neck region close to h29. (**C**) K^+^ ion stabilises the h18-uS12 interaction within the decoding centre. Hyp: 3-hydroxyproline. (**D**) Detail of K^+^ ion and water coordination within h28. (**E**) K^+^ ion bridges the eukaryote-specific ribosomal protein eS19 and the 18S rRNA. (**F**) K^+^ ion bridging eukaryote-specific eS8 and 18S rRNA. (**G**) Close-up of h18 within the body domain of the 18S rRNA showing detail of eukaryote-specific coordination of a K^+^ ion and water molecules with rRNA residues. (**H**) Detail of the solvation shell of h13 and the proximal h14, including a Mg^2+^ ion with octahedral coordination.

Water molecules, monovalent and divalent cations are important for ribosome structure and function (44–46). In our maps, we identified monovalent cations bound to both rRNA and RPs. We interpreted these as K^+^ ions given the composition of the buffers used during sample purification, together with their coordination geometry and distance. Interestingly, compared to the bacterial 30S ribosomal subunit (PDB ID: 6QNR) (44), the binding position of monovalent cations is conserved in key regions of the human 40S subunit. For example, we identified a K^+^ ion bound between h42 and uS13, which participates in formation of the neck-head junction within the head domain by coordinating the phosphate backbone of U1631 with the carbonyl groups of the backbone of residues Thr31, Ile33 and Val36 (Figure 4A). Similarly, in bacteria the protein backbone of uS13 is engaged in K^+^-mediated coordination with rRNA residue U1330; however, as the carbonyl group of the Ile25 backbone in bacteria does not face the ion binding pocket, it is therefore unable to interact with the K^+^ (Supplementary Figure S3A). Furthermore, in proximity to the modified residue m^7^G1639, we identified a K^+^ ion bound to the nucleobases of G1638, G1226 and G1227 and the phosphate backbone of G1226 with square anti-prismatic geometry (Figure 4B). An identical position for a K^+^ ion is found in bacteria (Supplementary Figure S3B). Human residue G626 (G530 bacterial numbering) on h18 is involved in the decoding mechanism, switching from a *syn*-conformation when the A-site is vacant, to the *anti*-conformation when tRNA is bound (47,48). Compared to the bacterial 70S ribosome (PDB: 6QNR), our model highlights a conserved K^+^ ion that interacts with residues C614 and G625 of h18, the hydroxyl group of the side chain of Asn63 and the carbonyl group of the 3-hydroxyproline at position 62 of uS12 (Figure 4C). Supporting our findings, in bacteria a K^+^ ion has also been experimentally assigned in this conserved pocket (Supplementary Figure S3C**)**. In our reconstructions, potassium ions mostly stabilise rRNA by coordinating stacked nucleotides, rather than by interacting with the phosphate backbone, consistent with data in bacteria (44). For instance, within h28, a K^+^ ion is involved in extensive coordination with rRNA nucleobases, that likely maintains base planarity (Figure 4D). Residue ψ1692 forms a non-complementary Watson-Crick base pair with G1206 in which the ψ1692 oxygen atom at position 4 directly interacts with the K^+^ ion. In bacteria, while the conserved uridine is unmodified (U1391), a K^+^ ion nevertheless establishes a similar bonding pattern (Supplementary Figure S3D).

In addition, we visualised K^+^ ions bound in eukaryote-specific positions. For example, we identified a K^+^ ion bound between the peptide backbone of the eukaryote-specific RP eS19 (residue Gly86) and the phosphate backbone of rRNA helices h30 and h42 (residues A1531 and G1606, respectively), which also interacts with two water molecules in square anti-prismatic coordination (Figure 4E). Similarly, in the three-way junction between helices h7, h11 and h12, a K^+^ ion bridged the 2’-OH group of A433, the oxygen at position 2 of U100 and the peptide backbone of eS8 (residue His22) (Figure 4F). We identified a K^+^ ion bound to the nucleobases of G602 and C603 at the bulge of h18, consistent with a key role in promoting rRNA stacking. Additionally, we identified two water molecules that coordinate the interaction between the nucleobase of A605 and the phosphate backbone of G_m_601 (Figure 4G). The evolution of the ribosome may have driven the requirement for ions. For example, on h13, rRNA residue C450 (C330 in *T. thermophilus*) adopts a different conformation in eukaryotes (Figure 4H, Supplementary Figure S3E), likely to allow access for the N-terminus of the eukaryotic specific ribosomal protein eS4. Interestingly, in the human map, a Mg^2+^ ion is bound with octahedral coordination to the phosphate backbone of C472, which establishes water-mediated contacts to the backbone and sugar moiety of G451 (Figure 4H). By contrast, in bacteria a K^+^ ion is found in this conserved position (Supplementary Figure S3E).

### Post-translational modifications of ribosomal proteins

**Figure 5.**
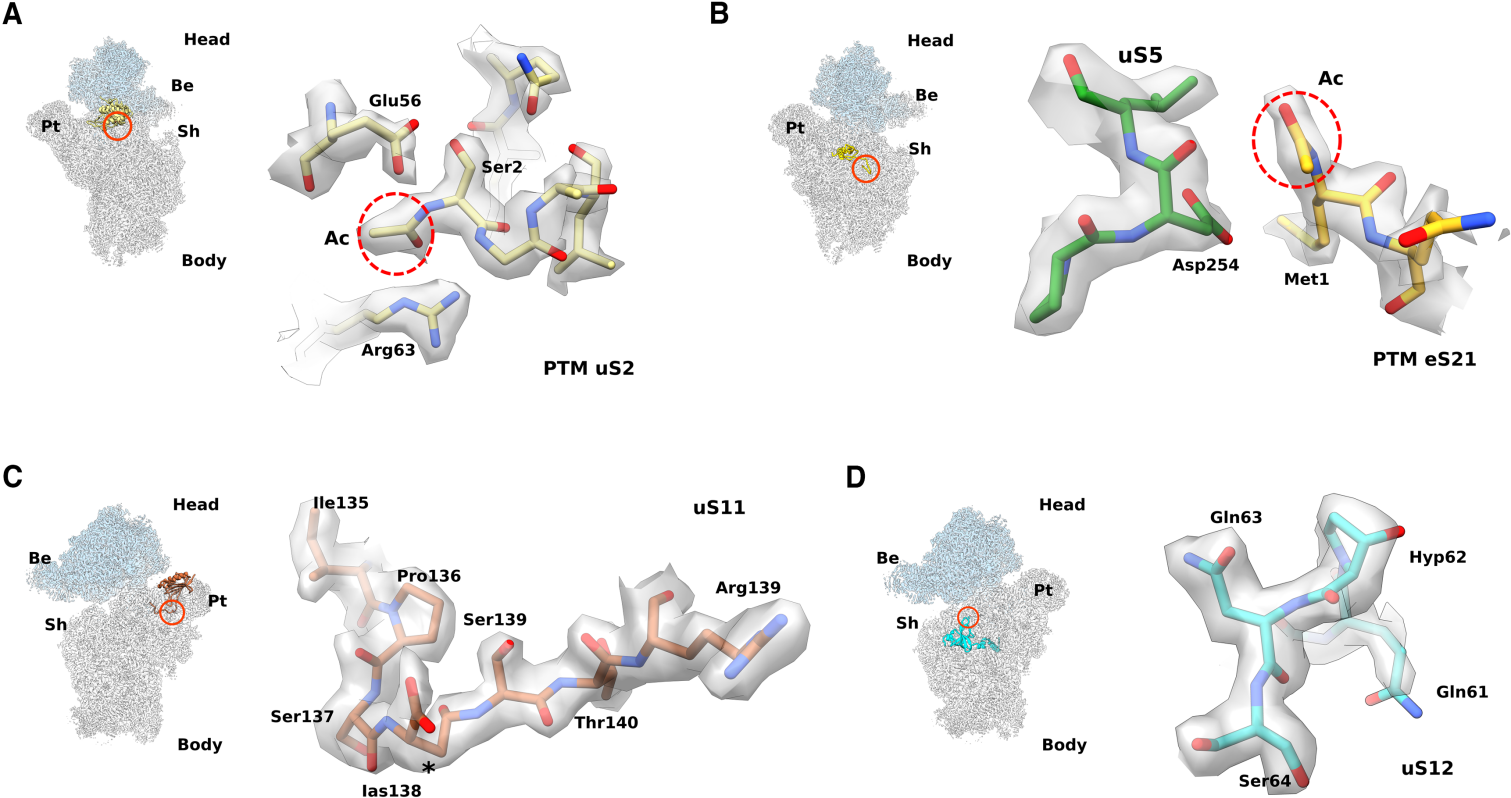
Visualising post-translational modifications of the human 40S subunit. Each panel contains the location of the ribosomal protein (in ribbon) where the PTM is identified. A red circle indicates the position of the modified aminoacid. (**A**) Close-up of the N-terminal region of the universally conserved ribosomal protein uS2. N-terminal acetylation of residue Ser2 is highlighted (red circle). (**B**) N-terminus of the eukaryotic specific eS21, with the N-terminal acetylation highlighted (red circle). (**C**) C-terminal region of uS11 fitted into the cryo-EM density. Asterisk indicates the extra methylene group of Ias138. (**D**) Close-up view of 3-hydroxyproline (Hyp) modification at position 62 of uS12. Be, beak; Pt, platform; Sh, shoulder.

Our cryo-EM reconstruction revealed the presence of additional density at the N-terminus of two ribosomal proteins, uS2 and eS21 (Figure 5A, B). uS2 and eS21 are part of the so-called S0-cluster and are among the last ribosomal proteins assembled into the 40S ribosomal subunit (49). Recruitment of the S0-cluster is thought to occur in the cytoplasm, after remodelling of the head domain and release of the biogenesis factor RRP12 (49,50). We hypothesised that the additional density might represent post-translational modifications, consistent with mass spectrometry data and a recent cryo-EM reconstruction of 80S ribosomes purified from rabbit reticulocytes (51–53). We therefore interrogated our human 40S sample for post-translational modifications using liquid chromatography mass spectrometry (LC-MS/MS) (Supplementary Table S3). Analysis of the tryptic peptides confirmed the presence of N-terminal acetylated uS2 on residue Ser2 and eS21 on residue Met1 (Supplementary Table S3). In addition, our MS analysis identified N-terminal acetylation of uS7, eS12, uS13 and eS24, in agreement with previous data combining bottom-up and top-down MS approaches (51); however, N-termini of these RPs were disordered, thus impairing their direct visualisation (Supplementary Table S3). Due to the absence of confirmatory tryptic peptides, we cannot exclude the possibility that additional 40S subunit ribosomal proteins may also be acetylated (36).

Our cryo-EM reconstruction revealed density corresponding to residue Asp138 of uS11 but incompatible with a canonical aspartate. Although the MS analysis encompassed Asp138, no post-translational modification was identified. We interpreted the extra density as an isoaspartyl residue (Figure 5C), consistent with cryo-EM analysis of the *E. coli* ribosome (46). In our structure, the isoaspartate (Ias) 138 side chain establishes a hydrogen bond with the cyclic oxygen of the ribose ring of rRNA residue G986 (Supplementary Figure S4A**)**. By contrast, the bacterial isoaspartyl residue (PDB: 7K00) does not interact directly with rRNA, but with Arg35 of the bacterial-specific protein bS21, while the neighbouring residue His118 and Glu31 from bS21 establish π-stacking and hydrogen bonding interactions, respectively, with A718 (Supplementary Figure S4A). Additionally, our cryo-EM density supports the modelling of 3-hydroxyproline at position 62 of uS12 (Figure 5D), in agreement with previous MS data (51) and a cryo-EM reconstruction of the rabbit 80S ribosome (53). We were unable to identify additional post-translational modifications in our reconstruction, such as lysine methylation, succinylation or acetylation, arginine methylation or hydroxylation or glycine myristoylation (dbPTM database: https://awi.cuhk.edu.cn/dbPTM/index.php) due to the low local resolution of specific residues (51,53) or possibly because some modifications, such as phosphorylation, may be dynamically incorporated in response to growth stimuli or cellular stress (54–56).

## DISCUSSION

The structural role of monovalent and divalent cations in ribosomal function has long remained a matter of debate. Although recent developments in long-wavelength X-ray diffraction allowed experimental assignment of K^+^ and Mg^2+^ in the bacterial ribosome (44), the high resolution that we have achieved in our reconstructions has allowed us to identify the position of ions directly and unambiguously in the most stable regions of the human 40S ribosomal subunit (Figure 4). Consistent with a role for Mg^2+^ ions in stabilising and promoting rRNA folding, we visualised about 90% of them bound to the phosphate backbone of the 18S with octahedral coordination. K^+^ ions instead bind to different elements of the ribosome to stabilise the interaction between a RP and rRNA via square anti-prismatic coordination (Figure 4A, C, E) or promote rRNA nucleobase stacking (Figure 4B, D, G). In several regions of the 40S ribosomal subunit, we observed K^+^ ions bound in identical positions compared to the bacterial ribosome, promoting similar bonding patterns (Figure 4A–D, Supplementary Figure S3A–D). In addition, we identified K^+^ ions bound in eukaryote-specific positions (Figure 4E–G), mediating similar structural roles.

RNA modifications are an important source of ribosome heterogeneity that may potentially modulate ribosome function and gene expression during normal development and in cancer (18,19,57–66). The human 40S ribosomal subunit alone contains 10 of the 14 identified rRNA post-transcriptional modifications (6), which are mainly found in functional regions such as the mRNA path, the decoding centre (DC) and the binding platforms for translation and assembly factors, as well as the core of the head and body domains, where they stabilise rRNA structure. Our reconstructions support the hypothesis that chemical modifications of rRNA fulfil structural and regulatory roles during ribosome biogenesis and translation. Of particular interest is the set of rRNA modifications within the h8-h14 region, where three 2’-*O*-methylations (C_m_462 and A_m_468, directly on h14; A_m_159, tip of h8) are incorporated (Supplementary Figure S5A). These modifications all lie within van der Waals radius distance, which may help promote h14 folding. In bacteria, mutations in residues within h8-h14 promote miscoding *in vivo* (67), attesting to the functional importance of this region for translation. In eukaryotes, h8-h14 forms the binding platform for several translation factors, such as the ATPase ABCE1 (involved in recycling) and the GTPases eIF5B and eIF1α (initiation), eEF2 (elongation) and eRF3 (termination). Due to its proximity to the nucleotide-binding domain of translation factors, the h8-h14 region may trigger nucleotide hydrolysis by these factors (48,68,69) (Supplementary Figure S5B). However, the underlying molecular mechanism remains elusive. In eukaryotes, the neck-head junction at h29 contains a conserved guanosine residue at position 1639, which carries an N^7^-methylation (Figure 2, Supplementary Figure S5C, D). During translocation, h29 participates in movement of the tRNA from the P- to the E-site, with residues G1639 and the neighbouring A1640 directly interacting with its anticodon stem loop (Supplementary Figure S5D).

Our cryo-EM reconstructions have allowed us to visualise post-transcriptional modifications in unprecedented detail (Figure 1–3). Achieving high-resolution maps of the 40S ribosomal subunit is a challenging task, given the presence of rRNA elements that are highly dynamic in absence of the large ribosomal subunit. In particular, h44, which constitutes the DC, spans the entire length of the body and a large portion of it is flexible when not interacting with H69-71 of the 60S ribosomal subunit. Nonetheless, our reconstruction allowed to visualise the unique rRNA modifications incorporated at the base of h44 (Figures 2 and 3). However, we were only able to visualise partial density for the hypermodified 1-methyl-3-(3-amino-3-carboxypropyl) ψ1248, confirming its high mobility when it is not bound to initiator tRNA (70,71) or to the assembly factor RIOK1 (43). Consistent with the SILNAS-based analysis of human 80S ribosomes (6), we visualised the previously unmodelled human rRNA modifications A_m_590, G_m_1447, ψ572, ψ863 and ψ1136 (Figure 1–3). Surprisingly, we were unable to validate one of the modifications recently identified by SILNAS-based quantification analysis, 2’-O-methylation of rRNA residue C621 (6), observing by contrast that the 2’-hydroxyl is bound to a structured water molecule (Figure 3B). We speculate that this discrepancy may relate to the different cells of origin for the 40S subunit samples (HEK293, this study and (43); TK6 and HeLa cells, (6)). However, more experimental evidence will be required to further test this hypothesis.

Our high-resolution reconstruction allowed us to visualise and propose a role for the N-terminal acetylation on uS2 and eS21 in stabilising interactions with neighbouring residues (Figure 5A–D). Furthermore, we have unambiguously traced the backbone of uS11 and identified a conserved isoaspartyl residue, consistent with recent findings in *E. coli* (46). Phylogenetic analysis of uS11 reveals a conserved C-terminal motif containing an asparagine in bacteria, but an aspartate in archaea and eukaryotes (46). We speculate that aspartate isomerisation in archaea and eukaryotes may promote rRNA folding through a direct interaction with the sugar ring of residue G986.

We have established a pipeline that enables cryo-EM reconstructions of human 40S subunits at resolutions close to 2 Å. We anticipate that this work will pave the way for high-resolution studies of human translation initiation complexes to explore the role of rRNA modifications and ion composition on ribosome function.

## DATA AVAILABILITY

Coordinates and cryo-EM density maps were deposited in the RCSB Protein Data Bank with accession code PDB 7R4X for the 40S ribosomal subunit model and the Electron Microscopy Database with accession codes EMD-14317, EMD-14318 and EMD-14319 for the entire human 40S, body and head domains, respectively. The mass spectrometric data were deposited to the ProteomeXchange Consortium via the PRIDE partner repository with the dataset identifier PXD031525.

## Supplementary Material

gkad194_Supplemental_Files

## Notes

Present address: Simone Pellegrino, Bicycle Therapeutics Ltd, Blocks A & B Portway Building, Granta Park, Great Abington, Cambridge CB21 6GS, UK.

Present address: Kyle C. Dent, Medical Research Council Laboratory of Molecular Biology, Cambridge CB2 0QH, UK.
