# Supplementary material for: Cryo-EM reconstruction of the human 40S ribosomal subunit at 2.15 Å resolution
Source: Nucleic Acids Res. 2023 Mar 23;51(8):4043–54. doi: 10.1093/nar/gkad194 (PMC10164566; doi:10.1093/nar/gkad194)
Supplement: gkad194_Supplemental_Files [file gkad194_supplemental_files.zip › Supplementary_material_revised_NAR.pdf]

Supplementary Figure S1

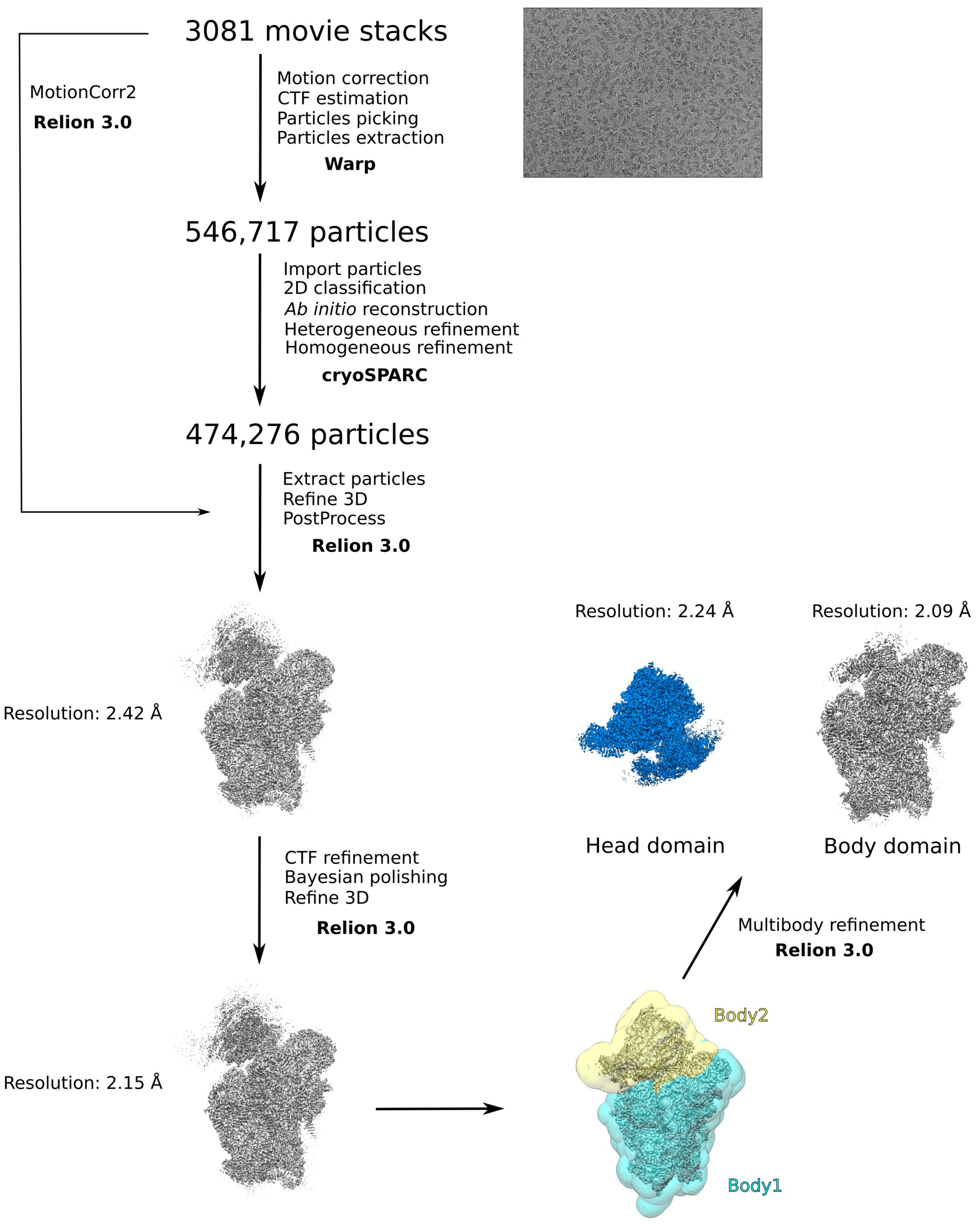

Supplementary Figure S1: Cryo-EM data processing scheme.

Supplementary Figure S2

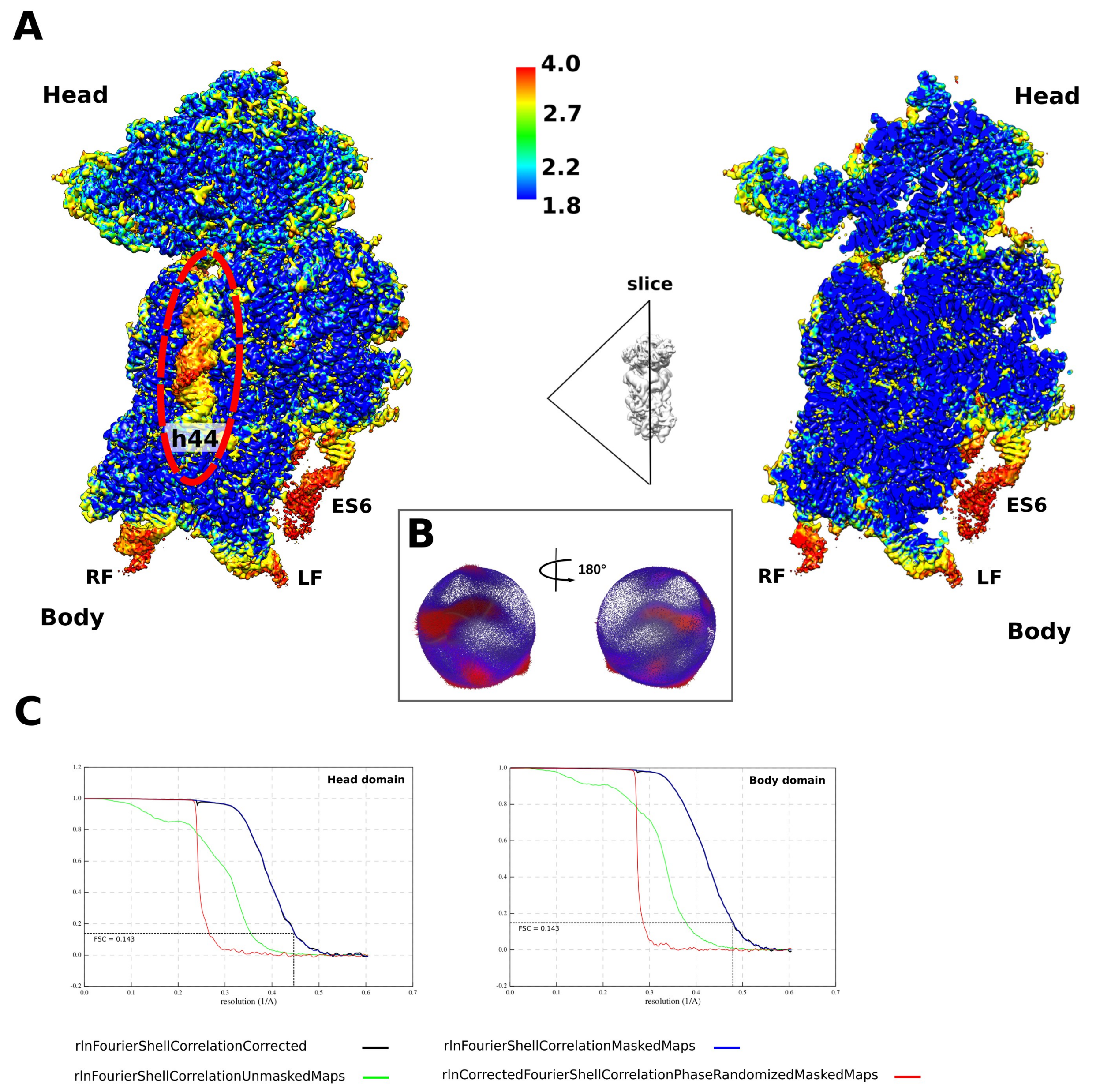

Supplementary Figure S2: Local resolution of human 40S subunit extends to 1.8 Å. A) Top left: local resolution estimate of the complete human 40S subunit, viewed from the intersubunit interface. The less well-resolved regions are indicated: h44 (red dashed line), right (RF) and left (LF) feet, rRNA expansion segment 6 (ES6). Top right: slice through the 40S showing high-resolution within the most stable regions of the 40S ribosomal subunit body and head domains. The colour scale indicates the resolution estimation values (Angstrom). B) Graphical representation of particle orientation distribution during 3D refinement (over-represented views in red). C) FSC

curves, as calculated within Relion 3.0 (28), for the head and the body domain. Gold-standard FSC cut-off at 0.143 to estimate the final resolution, is shown.

Supplementary Figure S3

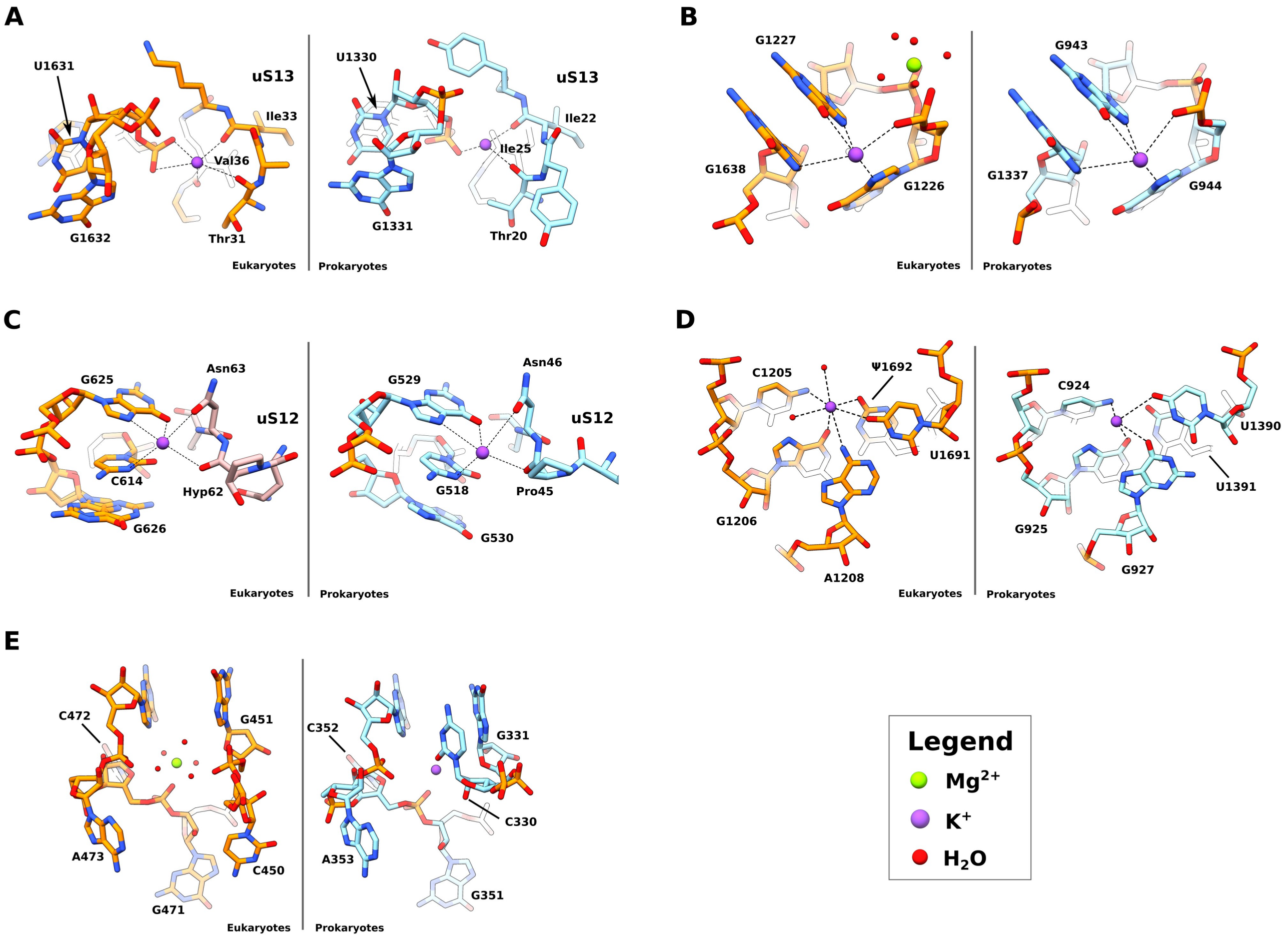

Supplementary Figure S3: Conserved 40S ribosomal subunit solvation. Comparison between human vs *Thermus thermophilus* small ribosomal subunit showing: A) K^+ ion stabilising the interaction between U1631 (bacterial U1330) on h42 with the universally conserved protein uS13; B) conserved potassium ion bound close to h29; C) close-up of the decoding centre showing a K^+ ion coordinating the interaction of h18 with the universally conserved ribosomal protein uS12; D) K^+ ion promoting nucleobases stacking on h28; E) eukaryote-specific binding of Mg^{2+} to the h13-14 region. *Thermus thermophilus* PDB 6QNR was used for comparison. Water molecules, K^+ and Mg^{2+} ions are shown as spheres (legend, bottom of panel).

Supplementary Figure S4

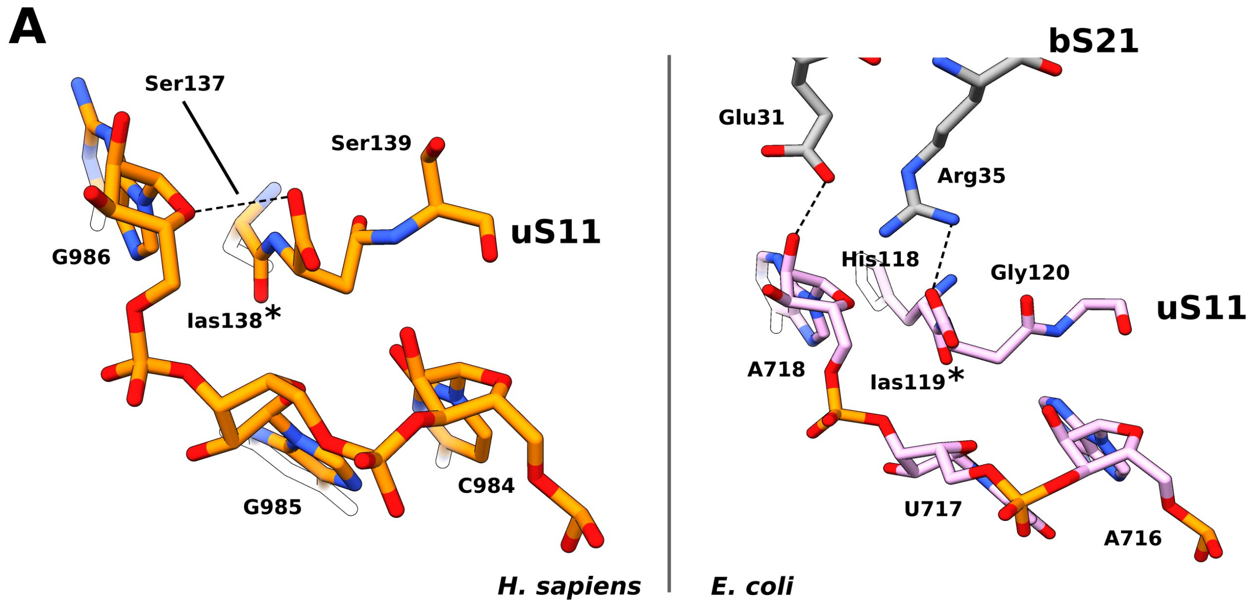

Supplementary Figure S4: Post-translational modifications may promote specific interactions.

A) Comparison of uS11 C-terminal residues and rRNA. *H. sapiens*, our model (left); *E. coli*, PDB: 7K00 (right).

Supplementary Figure S5

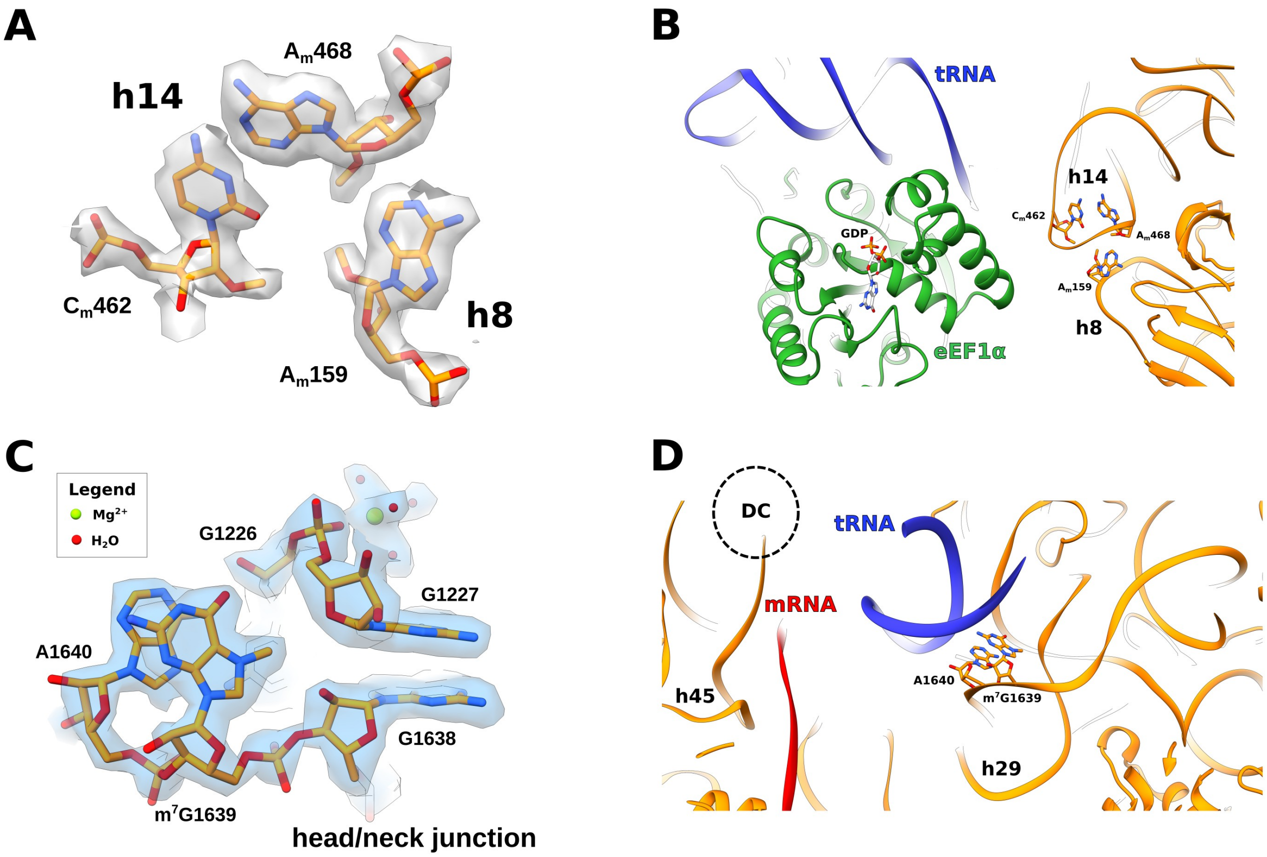

Supplementary Figure S5: rRNA modifications essential for translation. A) rRNA residues A_m159 (h8) A_m428 and C_m462 (h14) fitted into the cryo-EM density map of the 40S body. B) Close-up view of h8-h14 interaction with the eEF1α-tRNA complex (eEF1α in green, tRNA in blue). 18S rRNA residues A_m159, C_m462 and A_m468 and the GDP molecule within eEF1α are shown as sticks. C) Detail of the head/neck junction region on the head, with a close-up view of m^7G1639 and neighbouring rRNA residues, fitted within the cryo-EM density of the head. Water molecules and Mg^{2+} ions are shown as spheres (legend, top left). D) Interaction between rRNA residue m^7G1639 and P-site tRNA (in blue), upon superposition with human 48S initiation complex (PDB: 6ZMW). The mRNA (red) is shown for clarity.

Supplementary Table S1: Cryo-EM data collection, refinement and validation statistics.

	Human 40S (EMD-14317) (PDB 7R4X)	Head domain (EMD-14318)	Body domain (EMD-14319)
**Data collection and processing**			
Magnification	105,000x	105,000x	105,000x
Voltage (kV)	300	300	300
Electron exposure: total dose (e–/Å^2)	40.56	40.56	40.56
Defocus range (μm)	-2.8, -2.5, -2.2, -1.9, -1.6, -1.3, -1.0	-2.8, -2.5, -2.2, -1.9, -1.6, -1.3, -1.0	-2.8, -2.5, -2.2, -1.9, -1.6, -1.3, -1.0
Pixel size (Å)	0.83 (0.415 super-res)	0.83 (0.415 super-res)	0.83 (0.415 super-res)
Symmetry imposed	C1	C1	C1
Initial particle images (no.)	546,717	546,717	546,717
Final particle images (no.)	474,276	474,276	474,276
Map resolution (Å)			
FSC threshold	2.15	2.24	2.09
Map resolution range (Å)	4.00 – 1.80	4.00 – 1.80	4.00 – 1.80
Map sharpening B factor (Å^2)	-35.47	-38.64	-33.45
**Refinement**			
Initial model used (PDB code)	6G5H		
Model resolution range (Å)	5.00 – 2.15		
Model composition			
Non-hydrogen atoms	77936		
Protein residues	4792		
RNA bases	1643		
Ligands			
Water	4117		
Mg^{2+}	88		
K^+	82		
Zn^{2+}	3		
B factors (mean, Å^2)			
Protein	28.64		
RNA	30.86		
Ligand	24.06		
Water	21.96		
R.m.s. deviations			
Bond lengths (Å)	0.012		
Bond angles (°)	1.065		
Validation			
MolProbity score (percentile, 0-99 Å)	1.99 (76th)		
Clashscore (all atoms)	4.98 (94th)		
Poor rotamers (%)	4.09		
Ramachandran plot			
Favoured (%)	96.16		
Allowed (%)	3.78		
Disallowed (%)	0.06		
CaBLAM outliers (%)	2.10		
**Model vs. Map validation**			
CC_{mask}	0.85		
CC_{box}	0.80		
CC_{volume}	0.82		
FSC model-map (0.5) (Å)	2.12		
**RNA**			
Correct sugar puckers (%)	99.76		
Good backbone conformations (%)	84.00		

Supplementary Table S2: rRNA modifications modelled.

Residue numbering	Modification	Residue numbering	Modification
27	A_m	822	ψ
34	ψ	863	ψ
36	ψ	866	ψ
93	ψ	867	G_m
99	A_m	897	ψ
105	ψ	918	ψ
109	ψ	966	ψ
116	U_m	1004	ψ
119	ψ	1031	A_m
121	U_m	1045	ψ
159	A_m	1046	ψ
166	A_m	1056	ψ
172	U_m	1081	ψ
174	C_m	1136	ψ
210	ψ	1174	ψ
218	ψ	1177	ψ
296	ψ	1232	ψ
354	U_m	1238	ψ
406	ψ	1244	ψ
428	U_m	1248	$m^1acp^3\psi$
436	G_m	1272	C_m
462	C_m	1288	U_m
468	A_m	1326	U_m
484	A_m	1328	G_m
509	G_m	1337	ac^4C
512	A_m	1347	ψ
517	C_m	1367	ψ
572	ψ	1383	A_m
576	A_m	1391	C_m
590	A_m	1442	U_m
601	G_m	1445	ψ
609	ψ	1447	G_m
621	C_m	1490	G_m
627	U_m	1625	ψ

644	G_m	1639	m^7G
649	ψ	1643	ψ
651	ψ	1668	U_m
668	A_m	1678	A_m
681	ψ	1692	ψ
683	G_m	1703	C_m
686	ψ	1804	U_m
797	C_m	1832	m^6A
799	U_m	1842	ac^4C
801	ψ	1850	$m^6{}_2A$
814	ψ	1851	$m^6{}_2A$
815	ψ		

The following rRNA modifications were modelled:

Abbreviation	PDB name	Common name
ac^4C	4AC	N4-acetylcytidine
A_m	A2M	2'-O-methyladenosine
C_m	OMC	2'-O-methylcytidine
G_m	OMG	2'-O-methylguanosine
U_m	OMU	2'-O-methyluridine
$m^1acp^3\psi$	B8N	1-methyl-3-(3-amino-3-carboxypropyl) pseudouridine
$m^6{}_2A$	MA6	N6,N6-dimethyladenosine
m^6A	6MZ	N6-methyladenosine
m^7G	7MG	7-methylguanosine
ψ	PSU	Pseudouridine

Residues coloured in red were not built due to the lack of supporting cryo-EM density.
